# A questionnaire survey on radiation protection among 282 medical staff from 26 endoscopy‐fluoroscopy departments in Japan

**DOI:** 10.1002/deo2.5

**Published:** 2021-04-21

**Authors:** Shiro Hayashi, Mamoru Takenaka, Hirofumi Kogure, Takayuki Yakushijin, Hirotsugu Maruyama, Yasuki Hori, Toshiyuki Yoshio, Kenji Ikezawa, Tadayuki Takagi, Satoshi Asai, Kazuhiro Matsunaga, Kengo Matsumoto, Hidetaka Tsumura, Shinjiro Yamaguchi, Tetsuya Sumiyoshi, Koji Nagaike, Yuzuru Tamaru, Kazuo Hara, Toshio Fujisawa, Ichiro Oda, Ken Ohnita, Motohiko Kato, Hiroko Nebiki, Tatsuya Mikami, Akihiro Nishihara, Satoshi Egawa, Ryuki Minami, Makoto Hosono, Tsutomu Nishida

**Affiliations:** ^1^ Department of Gastroenterology and Internal Medicine Hayashi Clinic Osaka Japan; ^2^ Department of Gastroenterology Toyonaka Municipal Hospital Osaka Japan; ^3^ Department of Gastroenterology and Hepatology Kindai University Faculty of Medicine Osaka Japan; ^4^ Department of Gastroenterology The University of Tokyo Tokyo Japan; ^5^ Department of Gastroenterology and Hepatology Osaka General Medical Center Osaka Japan; ^6^ Department of Gastroenterology Osaka City University Graduate School of Medicine Osaka Japan; ^7^ Department of Gastroenterology and Metabolism Nagoya City University Hospital Aichi Japan; ^8^ Department of Gastroenterology Cancer Institute Hospital Tokyo Japan; ^9^ Department of Hepatobiliary and Pancreatic Oncology Osaka International Cancer Institute Osaka Japan; ^10^ Department of Gastroenterology Fukushima Medical University School of Medicine Fukushima Japan; ^11^ Department of Gastroenterology Tane General Hospital Osaka Japan; ^12^ Department of Gastroenterology Ishikawa Prefectural Central Hospital Ishikawa Japan; ^13^ Department of Grastroenterological Oncology Hyogo Cancer Center Hyogo Japan; ^14^ Department of Gastroenterology and Hepatology Kansai Rosai Hospital Hyogo Japan; ^15^ Department of Gastroenterology Tonan Hospital Hokkaido Japan; ^16^ Department of Gastroenterology and Hepatology Suita Municipal Hospital Osaka Japan; ^17^ Department of Gastroenterology Kure Medical Center and Chugoku Cancer Center Hiroshima Japan; ^18^ Department of Gastroenterology Aichi Cancer Center Aichi Japan; ^19^ Department of Gastroenterology Juntendo University Tokyo Japan; ^20^ Endoscopy Division National Cancer Center Hospital Tokyo Japan; ^21^ Department of Gastroenterology and Hepatology Shunkaikai Inoue Hospital Nagasaki Japan; ^22^ Department of Gastroenterology Keio University School of Medicine Tokyo Japan; ^23^ Department of Gastroenterology Osaka City General Hospital Osaka Japan; ^24^ Division of Endoscopy Hirosaki University Hospital Aomori Japan; ^25^ Department of Gastroenterology Minoh City Hospital Osaka Japan; ^26^ Department of Gastroenterology Osaka Police Hospital Osaka Japan; ^27^ Department of Gastroenterology Tenri Hospital Nara Japan; ^28^ Department of Radiology Kindai University Faculty of Medicine Osaka Japan

**Keywords:** education, endoscopy, medical staff, questionnaire survey, radiation protection

## Abstract

**Background and aims:**

It is essential for endoscopists, technologists, and nurses to understand radiation protection. However, protective equipment usage is still low, and there is little awareness of radiation protection in practice.

**Methods:**

We conducted a questionnaire survey on radiation protection from January to February 2020. The participants were medical staff, including medical doctors, nurses, and radiological and endoscopy technician in endoscopy‐fluoroscopy departments. The questionnaire included 14 multiple‐choice questions divided among three parts: background, equipment, and knowledge.

**Results:**

We surveyed a total of 282 subjects from 26 institutions. There were 168 medical doctors (60%), 90 nurses (32%), and 24 technologists (9%). Although almost all staff members (99%) always wore a lead apron, only a few wore a thyroid collar (32%) and lead glasses (21%). The rate of wearing a radiation dosimeter was insufficient (69%), especially among doctors (52%). A few subjects knew the radiation exposure dose of each procedure (15%), and slightly over half had attended lectures on radiation protection (64%) and knew about the three principles of radiation protection (59%). Protection adherence did not differ by years of experience, knowledge of fluoroscopy, awareness of radiation exposure doses, or attendance at basic lectures on radiation protection. However, medical doctors who were aware of the radiation exposure dose of each procedure were significantly more likely to wear dosimeters than those who were not (p = 0.0008).

**Conclusion:**

Medical staff in endoscopy departments in Japan do not have enough radiation protection equipment or education.

## INTRODUCTION

Radiation protection is the basis for the safety of both patients and medical staff due to its adverse effects represented by carcinogenicity and skin disorder.^1^, ^2^ The International Commission on Radiological Protection (ICRP) stated that an understanding and awareness of the hazards of radiation among medical staff can prevent unnecessary risks for the population as a whole.^3^, ^4^ In the field of gastroenterology, the World Gastroenterology Organization practice guidelines and the European Society of Gastroenterology Endoscopy guidelines state the importance of radiation protection.^5^, ^6^ However, some reports from Ireland, Korea, and the United States still showed low protective equipment usage and little awareness of radiation protection in practice.^7^, ^8^, ^9^ The use of protective equipment and awareness of radiation protection do not appear to be widespread. Currently, we are conducting a prospective multicenter study in Japan (REX‐GI study, UMIN000036525) that will involve the collection of actual radiation exposure‐related data from digestive endoscopy interventional procedures.^10^ To complement this study, we conducted a nationwide questionnaire survey to assess the actual rate of equipment usage and knowledge and awareness of radiation protection among staff at institutions related to the REX‐GI study group and the Fight Japan study group, including gastroenterology doctors, nurses, and technologists from endoscopy‐fluoroscopy departments in Japan.

## MATERIALS AND METHOD

We conducted a questionnaire survey on radiation exposure protection and collected responses from January 2020 to February 2020. We emailed a representative doctor in each institution involved in the REX‐GI study^10^ and Fight Japan study group, as well as at other institutions in Japan and invited them to participate in the questionnaire survey. If the representative accepted our invitation, he or she also asked the medical staff, doctors, nurses, and radiological and endoscopy technicians from the fluoroscopic endoscopy suites at each institution to answer an anonymous online questionnaire using Google Forms. Endoscopy technician is a job to maintain the endoscopic equipment and to support the endoscopic procedures in the endoscopy unit. Participants provided informed consent by opening the survey.

The questionnaire used in the survey included 14 multiple‐choice questions divided among the following three parts: background, equipment, and knowledge. The details of the questionnaire details are shown in Table 1. Briefly, questions 1–6 regard the background of each person or institution. Questions 7–10 asked about the proper equipment for radiation protection. Questions 11–14 focused on knowledge of radiation exposure and protection. We counted the number of responses from subjects with each job title and compared the numbers of responses among job titles. We then investigated how years of career experience, facility size, and knowledge influenced protective behaviors among doctors.

**TABLE 1:** Questionnaire questions and answers (participants’ responses were anonymous)

Question	Answer
1. What is your gender?	a) Female, b) Male
2. How old are you?	a) Twenties, b) Thirties, c) Forties, d) Fifties, e) Over sixty
3. What is your job title?	a) Medical doctor, b) Nurse, c) Technologist
4. What is the size of your institution?	a) University hospital or medical center, b) Regional general hospital (>300 beds), c) Other
5. How many years of career experience do you have?	a) 1–5, b) 6–10, c) 11–15, d) 16–20, e) Over 21 years
6. Do you operate the fluoroscopy unit?	a) Yes, b) No
7. Do you always wear a lead apron?	a) Yes, b) No
8. Do you always wear a thyroid collar?	a) Yes, b) No
9. Do you always wear lead glasses?	a) Yes, b) No
10. Do you always wear a radiation dosimeter?	a) Yes, b) No
11. What type is your fluoroscopy unit, an under‐couch or over‐couch C‐arm system?	a) Under‐couch (exposure from below), b) Over‐couch (expose from above), c) I don't know
12. Do you know how much radiation dose you are exposed to in each endoscopic procedure under fluoroscopy?	a) Yes, b) No
13. Have you ever attended a basic lecture on radiation exposure?	a) Yes, b) No
14. Do you know the three principles of radiation protection?	a) Yes, b) No

### Statistical analysis

The categorical variables are expressed as the number in each category or the frequency and were compared using the chi‐square test or Fisher's exact test when appropriate. A p‐value of 0.05 was considered to indicate statistical significance. All statistical analyses were performed with JMP software (ver. 14.3, SAS Institute Inc., Cary, NC, USA).

## RESULTS

### Responses to the questionnaire

We emailed survey invitations to one hundred and eleven institutions. We obtained answers from two hundred eighty‐two subjects, including endoscopists, nurses, and technicians at 26 institutions (participation rate: 26/111, 23.4%).

Questions 1–6: There were 166 (59%) males. Most of the subjects were in their 30s (105, 37%) or 40s (103, 37%). Doctor was the most common occupation (168, 60%). A total of 135 subjects worked at university hospitals or medical center hospitals (48%), 110 worked at regional general hospitals (>300 beds) (39%), and the other 37 worked at other types of hospitals (13%). Regarding years of experience, 45 (16%) had 1–5 years and 68 (24%), 56 (20%), and 56 (20%) had 6–10, 15‐20, and over 21 years, respectively. One hundred eighty‐eight subjects (67%) had operated fluoroscopy units by themselves (Table 2). Questions 7–10: Two hundred eighty‐one subjects (99%) always wore a lead apron, 90 subjects (32%) wore a thyroid collar, 59 subjects (21%) wore lead glasses, and 194 subjects (69%) wore a radiation dosimeter. Questions 11–14: Thirty subjects (11%) did not know the type of fluoroscopy unit. Forty‐two subjects (15%) were aware of the radiation dose of each procedure, 180 subjects (64%) had received lectures on radiation protection, and 167 subjects (59%) were aware of the three principles of radiation protection (Table 2)

**TABLE 2:** Answers for all subjects including medical doctors, nurses, and technologists

Questions	Answer	All N = 282	Medical doctors N = 168	Nurses N = 90	Technologists N = 24
1. Sex, N (%)	Male	166, 59%	144, 86%	8, 9%	14, 58%
	Female	116, 41%	24, 14%	82, 91%	10, 42%
2. Age group, N (%)	20–29	45, 16%	17, 10%	18, 20%	10, 42%
	30–39	105, 37%	69, 41%	27, 30%	9, 38%
	40–49	103, 37%	71, 42%	29, 32%	3, 13%
	50–59	26, 9%	10, 6%	14, 16%	2, 8%
	60 and over	3, 1%	1, 1%	2, 2%	0, 0%
3. Job title		282	168, 60%	90, 32%	24, 9%
4. Institution size	University hospital or medical center	135, 48%	86, 51%	41, 46%	8, 33%
	Regional general hospital (>300 beds)	110, 39%	17, 10%	32, 36%	13, 54%
	Other	37, 13%	65, 39%	17, 19%	3, 13%
5. Career experience, years	1–5	45, 16%	24, 14%	12, 13%	9, 38%
	6–10	68, 24%	40, 24%	20, 22%	8, 33%
	11–15	56, 20%	37, 22%	16, 18%	3, 13%
	16–20	57, 20%	40, 24%	16, 18%	1, 4%
	Over 21	56, 20%	27, 16%	26, 29%	3, 13%
6. Operation of the fluoroscopy unit	Yes	188, 67%	156, 93%	23, 26%	9, 38%
7. Use of lead apron	Yes	281, 99%	168, 100%	89, 99%	24, 100%
8. Use of thyroid collar	Yes	90, 32%	46, 27%	35, 39%	9, 38%
9. Use of lead glasses	Yes	59, 21%	35, 21%	18, 20%	6, 25%
10. Use of radiation dosimeter	Yes	194, 69%	87, 52%	85, 94%	22, 92%
11. Fluoroscopy unit type	I don't know	30, 11%	11, 7%	17, 19%	2, 8%
12. RE of each procedure	Yes	42, 15%	21, 13%	12, 13%	9, 38%
13. Basic lecture on RE	Yes	180, 64%	119, 71%	49, 54%	12, 50%
14. Three principles of RP	Yes	167, 59%	102, 61%	53, 59%	12, 50%

Abbreviations: RE, radiation exposure; RP, radiation protection.

### Differences according to job title

**FIGURE 1:**
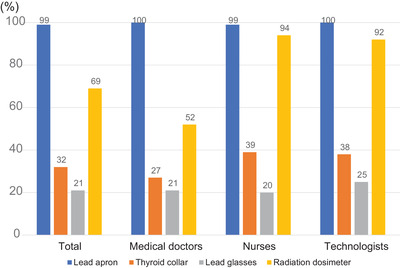
Rates of use of protective equipment for radiation exposure according to job title

Of the medical doctors, one hundred fifty‐six (93%) had directly operated fluoroscopy units. By contrast, fewer nurses (N = 23, 26%) and technologists (N = 9, 38%) had operated fluoroscopy units because nurses and technologists are not licensed to manipulate radiation equipment except when instructed by an attending physician or dentist in Japan. The rates of wearing a lead apron among medical doctors, nurses, and technologists were 100% (N = 168), 99% (N = 89), and 100% (N = 24), respectively. Compared with the rates of wearing a lead apron, the rates of wearing a thyroid collar were low among medical doctors (27%, N = 46), nurses (39%, N = 35), and technologists (38%, N = 9). Similarly, the rates of wearing lead glasses were also low among in medical doctors (21%, N = 35), nurses (20%, N = 18), and technologists (25%, N = 6). Rates of wearing radiation dosimeters were 52% (N = 87) by medical doctors, 94% (N = 85) by nurses, and 92% (N = 22) by technologists (Figure 1). Medical doctors were significantly less likely to wear dosimeters than the other medical workers (p < 0.0001). Regarding knowledge of radiation protection, 11 medical doctors (7%), 17 nurses (19%), and two technologists (8%) were not aware of the type of fluoroscopy unit. In total, 21 medical doctors (13%), 12 nurses (13%), and nine technologists (38%) were aware of the radiation dose of each procedure. One hundred nineteen medical doctors (71%), 49 nurses (54%), and 12 technologists (50%) had received lectures on radiation protection. In total, 102 medical doctors (61%), 53 nurses (59%), and 12 technologists (50%) were aware of the three principles of radiation protection.

### Factors affecting questionnaire answers

Use of equipment for radiation protection, including a lead apron, a thyroid collar, lead glasses, and a radiation dosimeter, did not differ based on years of experience. Similarly, the subjects did not differ in their recognition of the types of fluoroscopy; awareness of the radiation exposure dose of each procedure; attendance at basic lectures on radiation protection, including on the three principles of radiation protection; and size of the facility. However, medical doctors who were aware of the radiation exposure dose of each procedure wore dosimeters significantly more than those who were not (p = 0.0008). Medical doctors who had received basic lectures on radiation exposure were significantly more aware of the three principles of radiation protection (p < 0.0001), and those who were aware of the three principles were significantly more likely to have received lectures on radiation exposure (p < 0.0001).

## DISCUSSION

This large Japanese questionnaire survey revealed that most of the medical staff in endoscopy departments usually wore lead aprons (almost 100%) but that they did not always wear thyroid collars (27%), lead glasses (21%), or dosimeters (52%) (Table 2, Figure 1). A previous Korean questionnaire survey conducted in 2011 showed similar adherence rates of endoscopists (aprons: 100%, thyroid collars: 52%, lead grasses: 14%, dosimeters 10%).^8^ Our cohort had slightly better rates of wearing dosimeters, but the rates were still insufficient. A recent document by the Ministry of Health, Labor, and Welfare of Japan reported that endoscopists had a lower dosimeter equipment usage rate than interventional radiologists (43% vs 100%).^11^ Moreover, the rate of the usage of lead glasses by endoscopists in the current study was extremely low (21%). The authors of the previous study reported that medical doctors and nurses in the gastroenterology department had much higher doses of radiation exposure to the lens than those of other departments, in which 11% of doctors and 50% of nurses received radiation exposure amounting to greater than 20 mSv/year.^11^ Above all, the lack of knowledge of lens exposure causes a lower adherence rate for the use of lead glasses. There is an urgent necessity to gather information about global efforts, such as the revision of the upper limit of lens exposure doses in recent years.^12^ The significant difference between the fields of radiology and cardiology and the field of gastroenterology may depend on the provision of academic lectures led by academic societies.

The rate of dosimeter use was 69% among all subjects. However, the rate of wearing a dosimeter among medical doctors (52%) was much lower than among nurses (92%) and technologists (94%), which led to lower overall adherence (Table 2, Figure 1). Campbell et al reported in 2002 that 47% of endoscopists performing ERCP never used dosimeters.^13^ Similarly, a Korean study from 2011 reported a rate of dosimeter use of 10%.^8^ This previous study showed that poor adherence has not improved for nearly two decades, which may reflect the situation in Japan, even though the previous survey was conducted in a different country. Radiation monitoring is also essential for basic fluoroscopic guidance practices that must be conducted to minimize exposure doses. However, only 21 doctors (13%) were aware of the radiation exposure dose of each procedure in the present survey.

The ICRP has stated the importance of radiation protection knowledge and education.^4^ However, there are still many reports of doctors’ low awareness of radiation protection.^14^ Dauda et al reported that 80% of doctors had not attended basic lectures about radiation protection.^15^ Sethi et al conducted a questionnaire survey of 159 endoscopists in the United States and reported that the majority of endoscopists (62%) directly performed fluoroscopy during ERCP but that 57% had not received lectures on operating fluoroscopy equipment.^9^ Similarly, we believe that it is problematic that among the greater than 90% of the endoscopists in the present survey who operated fluoroscopy equipment, as only 71% took the basic courses. There have been many reports that education is useful for reducing radiation exposure. Specifically in the field of pediatric computed tomography, education and training programs for radiological institutes were concluded to be effective in achieving a substantial reduction in the radiation exposure dose.^16^, ^17^, ^18^ In the field of cardiovascular medicine, Georges et al demonstrated in 2009 that training in radiation protection for interventional cardiologists was associated with a 50% reduction in radiation exposure.^19^ In addition, sustained practice and X‐ray system changes can result in a 40% decrease in radiation exposure.^20^ In the field of gastroenterology, Barakat et al reported the effectiveness of brief education for radiation protection.^21^ In the present survey, years of experience and the size of the facility did not affect the use of radiation protection, indicating that even experienced staff in high‐volume center had not received enough education (Table 3). Our recent study revealed that awareness and education might reduce radiation exposure.^22^ We believe that the solution is to create an environment where education is widely available to both experienced and novice medical staff in endoscopy units; for example, mandatory educational lectures at conferences, such as those of radiological societies, may be considered.

**TABLE 3:** Factors affecting doctors’ questionnaire responses

Questions	Years of experience	Size of facility	RE in each procedure	Basic lecture on RE	Three principles of RP
Lead apron (yes)	NSD	NSD	NSD	NSD	NSD
Thyroid collar (yes)	NSD	NSD	NSD	NSD	NSD
Lead glasses (yes)	NSD	NSD	NSD	NSD	NSD
Radiation dosimeter (yes)	NSD	NSD	p = 0.0008	NSD	NSD
Type of fluoroscopy unit (I don't know)	NSD	NSD	NSD	NSD	NSD
RE in each procedure (yes)	NSD	NSD	–	NSD	NSD
Basic lecture on RE (yes)	NSD	NSD	NSD	–	p < 0.0001
Three principles of RP (yes)	NSD	NSD	NSD	p < 0.0001	–

Abbreviations: RE, radiation exposure; RP, radiation protection; NSD, no significant difference.

In conclusion, this nationwide multicenter questionnaire survey of 282 medical staff showed the current status of protective equipment usage, awareness, and education in endoscopy departments in Japan. At present, the low rate of dosimeter wearing among gastroenterologists is a major problem, and there may be a lack of education in the gastrointestinal field in Japan. Continuing education can solve these problems, and endoscopists must be aware of the importance of radiation protection to protect both patients and staff. In addition, we are currently conducting a prospective nationwide study in Japan (REX‐GI study) to collect actual radiation exposure data during digestive endoscopy. After this study, we plan to conduct a second questionnaire study to survey changes over the period between the two surveys.

## CONFLICT OF INTEREST

The authors report no conflict of interest. The authors alone are responsible for the content and writing of this paper.

## ETHICS DECLARATIONS

All participants were informed about the study. After giving their informed consent by checking the agree box in the web‐based questionnaire, the participants were enrolled in the study. The study was performed following the guidelines of the declaration of Helsinki. Ethical approval was not sought for the present study because of the anonymous questionnaire survey.

## FUNDING INFORMATION

This research received clinical research grants from the Japanese Society of Gastroenterology.
